# Qualitative Evaluation of a Comprehensive Online Wellness Program (MENTOR) Among People With Spinal Cord Injury

**DOI:** 10.3389/fresc.2022.917898

**Published:** 2022-06-10

**Authors:** James H. Rimmer, Jereme Wilroy, Hui-Ju Young, Raven Young, Tanvee Sinha, Madison Currie, Carla Rigo Lima, Byron Lai

**Affiliations:** ^1^Dean's Office, University of Alabama at Birmingham, Birmingham, AL, United States; ^2^University of Alabama at Birmingham-Lakeshore Foundation Research Collaborative, Birmingham, AL, United States; ^3^Department of Physical Medicine and Rehabilitation, University of Alabama at Birmingham, Birmingham, AL, United States; ^4^Division of Pediatric Rehabilitation Medicine, Department of Pediatrics, University of Alabama at Birmingham, Birmingham, AL, United States; ^5^Physical and Occupational Therapy Department, Rehabilitation Science Program, University of Alabama at Birmingham, Birmingham, AL, United States

**Keywords:** wellness, disability, exercise, nutrition, lifestyle program, telehealth

## Abstract

People with spinal cord injury (SCI) experience a plethora of health conditions that hinder their health and wellness. This qualitative retrospective evaluation describes the perceptions of 14 peoples with SCI, several months after they completed an eight-week telewellness community program (MENTOR—Mindfulness, Exercise and Nutrition To Optimize Resilience). The program offered daily online classes that covered three core wellness domains (mindfulness, exercise, nutrition) and one health coaching session to introduce participants to eight other wellness domains (sleep, self-care, core values, arts & leisure, outdoor time in nature; spiritual practice, relationships, contribution to others). Qualitative analysis resulted in 4 themes related to program benefits, likes, and improvement recommendations. First, participants valued the program for the social support provided by a sense of community and relationship building with peers. Second, self-regulation was facilitated by the comprehensiveness of the program components, easy online access, and shared lifestyle goals for self-improvement among peers. Third, participants reported improved psychological wellbeing and adopted healthy behaviors that were maintained long after the program. Last, future programs should include flexible class times, post-program support, specific exercise adaptations for people with limited arm function, and supplementary in-person meetings. These preliminary findings demonstrate that MENTOR may benefit the wellbeing of people with SCI and warrant further study.

## Introduction

The connection between healthcare and wellness is typically not available in conventional healthcare systems in the United States and most other parts of the world (1). With reference to the U.S., we have yet to address the societal responsibilities and barriers that prevent individuals with spinal cord injury (SCI) from leading healthy, active lives. Simply put, we have not achieved health equity. Working-age adults with SCI visit their healthcare provider and the emergency department more frequently than people without disability (2), and individuals with SCI—both recently acquired and existing for many years—are seldom exposed to wellness programs that focus on improving their quality of life and helping them prevent or manage physical and psychosocial secondary health conditions (1). The secondary conditions associated with disability, overlapping the natural course of aging, can bring an onset of new medical issues across an individual's lifespan (3–6).

Unhealthy behaviors such as a poor diet and lack of exercise can predispose people with SCI to even greater levels of adverse health consequences when they overlay such secondary conditions as pain (neuropathic and musculoskeletal) (7, 8), excess weight gain (i.e., obesity) (9–12), and deconditioning (i.e., extremely low fitness levels and high levels of sedentary behavior) (13–15). These health conditions have a profound negative impact on *health and function* and in the aggregate, can impose substantial limitations in rates of participation in general life activities including employment, social and community engagement and performing instrumental activities of daily living.

Expertise, support, and resources to address specific health issues or measures to prevent them are difficult to find, particularly in key areas of integrative wellness that include exercise, nutrition, mindfulness, sleep, and other less known, but important health-enhancing behaviors (16, 17). This may leave many people with SCI underprepared to return to their community and self-manage their health. The lack of continuity between the completion of rehabilitation and the initiation of wellness is clearly not helping the situation (18).

Physicians may not feel comfortable addressing certain physical or cognitive conditions associated with the disability (19), which adds to the challenge of patients with disability moving beyond medical care into self-managed wellness (3). Studies demonstrate that people with SCI experience issues with depression, anxiety, post-traumatic stress disorder, life-satisfaction, and overall quality of life (20).

People with SCI are also at increased risk for developing chronic health conditions at a much higher rate than the general population, including heart disease, obesity, and type 2 diabetes (21). These conditions may result from a lack of poor access to wellness programs that include physical activity, nutritional instruction and mind-based stress reduction (22). For these reasons, identifying and/or developing effective models for preventing and treating secondary conditions in people with SCI continues to be an important priority (23).

There is a growing body of evidence that convincingly supports the effectiveness and superiority of non-invasive wellness interventions that are consistent with contemporary lifestyle medicine in the promotion of lifelong health and wellness (16, 24). Understanding how to expand the continuum of healthcare services into the domains of wellness to allow individuals with SCI to self-manage their health and maintain adequate resilience continues to be a strong need in SCI research (5, 25–27). The SCI literature is starting to recognize the importance of resilience (20, 28, 29), which refers to the person's ability to understand, cope, adapt and strive for a positive balance between gains and losses in health and function across their lifespan.

The MENTOR program (Mindfulness, Exercise, and Nutrition To Optimize Resilience) has been offered to people with SCI and other physical disabilities across the U.S. for the past year. MENTOR is a community project (i.e., not a clinical trial) funded by the Center for Disease Control and Prevention. The purpose of this retrospective evaluation was to examine the perceptions of a subset of people with SCI who completed MENTOR, with a specific focus on program benefits and likes and dislikes related to the implementation of the program, with the intent to use these findings to refine the next iteration of MENTOR.

## Methods

### Design

This was a retrospective, qualitative program evaluation to understand the end users' perspective from which to make future modifications to the program. The philosophical assumptions that underpinned all phases of the research were critical realism ontological perspective (i.e., reality is multiple and subjective) and an interpretivism epistemological perspective (i.e., knowledge is socially constructed by both the participant and researcher). Put simply, the research time acknowledged that bias from participants and the team is unavoidable and should be considered throughout the study. Participants experienced different perspectives of the program, and recollections of the program could be heavily influenced by the interaction between the interviewer and analysts, as well as the presentation of the results. The team agreed upon the philosophical assumptions and aimed to incorporate only a minimal level of bias/interpretivism when conducting all aspects of the project.

### MENTOR Program

The MENTOR program is an 8-week, 40-h online telewellness program created for people with SCI and other physical disabilities with the aim to provide wellness support. The program consists of 11 evidence-based wellness domains that have been proven effective for improving health (16, 30). These include exercise, diet, sleep, self-care, mindfulness, core values, arts & leisure, outdoor time in nature; spiritual practice, relationships, contribution to others. The exercise, nutrition and mindfulness classes are considered the *core* domains because they are offered weekly as hands-on sessions taught by qualified specialists in these respective fields. The remaining wellness domains are offered in a 1-h session, once per week, across the 8-week program. Further details of the program can be found elsewhere (31).

#### MENTOR Core Wellness Domains

##### Exercise

The exercises classes were offered 2 days a week (Tuesday/Thursday) for 1 h each day, and each class was split into 30 min of exercise form and safety, followed by 30 min of performing the exercise. The fitness activities were adapted based on the participants' functional level. Participants with SCI performed the exercise routines from a seated position.

##### Nutrition

The nutrition classes were taught once a week (Wednesday) by a registered dietitian and focused on preparing meals, learning new recipes that focused on increasing fruit and vegetable consumption, and budgeting groceries to include healthier options and reduce processed foods. All the recommended foods were approved by the Women and Infants/Children (WIC) and Supplemental Nutrition Assistance Program (SNAP) U.S. federal assistance programs.

##### Mindfulness

The mindfulness classes were taught once a week (Monday) by a trained instructor who emphasized various aspects of traditional mindfulness techniques that included comfortable positioning techniques for those in a wheelchair, proper breathing control, and strategies to combat negative feelings or psychological issues.

#### MENTOR Wellness Sub-domains

The eight sub-domains included self-care skills, core values, outdoor time in nature, sleep, contribution to others, arts and leisure, relationships, and spiritual practice. Participants practiced these domains once a week (Friday) with their assigned groups and trained health coaches, who emphasized the “high level pointers” for improving each of these domains. For example, under sleep, various suggestions were provided based on the sleep literature to improve sleep hygiene. These included use of a white or pink noise machine, maintaining an ideal room temperature (64–66 degrees), using a weighted blanket, increasing room darkness, avoiding blue light from cell phones, going to bed and waking up at a regular time, along with several other suggestions. These sessions were meant to help participants understand how to self-manage their health within these domains, and independently seek and utilize resources to improve any wellness domain that they were having difficulty with or wanting to learn more about its potential benefit for improving their health.

#### Recruitment/Eligibility

This study aimed to recruit a convenience sample of 20 participants or recruit participants until surface-level themes were *saturated* (i.e., themes that appropriately and adequately address the research questions) (32). Participants with SCI who completed the MENTOR program were recruited for this study. Eligibility criteria included: (1)18–70 years of age; (2) internet access; and (3) ability to access the program materials independently.

#### Procedures

The entire MENTOR program was conducted remotely and included no in-person interactions. Participants were arranged in groups of 6–10 participants. The program was offered in waves, and 2 weeks prior to the start of each wave, the participant coordinator recontacted participants to confirm their interest and to provide details on their schedules, equipment, etc. Weekly check-ins were conducted with participants as reminders for upcoming classes for the week and what materials they should read or complete (i.e., a worksheet on a certain wellness domain) prior to the session. If a participant did not show up for class and did not notify their health coach in advance, he/she was called to make sure everything was ok and to document the reason for missing the class. Participants were asked to attend all of the classes through Zoom videoconference software. They were able to interact or contact their health coach or instructors prior to or after the class if they had any questions or a specific issue. They were sent a small set of inexpensive exercise equipment (e.g., hand weights, elastic bands) that was shipped to their home prior to the start of the program. They were also sent a MENTOR journal and were asked to record various activities that they did during the week that related to one or more wellness domains.

At the beginning of the program, participants were provided with program logistics, and were introduced to MENTOR's web-based commercial platform (Healthie). Because the program was completely online, there was a telelogistics support protocol in place. MENTOR components, such as online enrollment, assessment, and implementation were managed by a telelogistics support team. This was made up of four program staff and six health coaches. The local institutional reviewer board of the University granted a research exemption for this study designating it as a quality improvement evaluation study of an existing program.

Several months after completing MENTOR, participants were contacted *via* telephone to participate in a program evaluation interview. The interview lasted no longer than 30 min and included ~10 questions. The questions were semi-structured and asked participants about their overall thoughts of the program, likes/dislikes, potential benefits, and general questions pertaining to the 3 core components of the program (exercise, mindfulness, and nutrition). The questions also included a general ice-breaker question (asking about what the participant likes to do in their free time), and a question asking what recommendations they would have for improving the program. The interviews were conducted by three research staff who had several years of experience in wellness-related fields for people with disabilities. None of the interviewers were involved with the delivery of MENTOR. The interviewers were instructed to ask follow-up questions to clarify and expand on noteworthy topics that participants reported. Interviews were audio recorded and transcribed by a 3^rd^ party transcription company. Participants were thanked with a $20 electronic debit card for completing the interview.

### Analysis

Inductive thematic analysis (33) was used to analyze qualitative data. First, the research team transcribed and crosschecked the data for accuracy while reading the transcriptions. Next, two analysts generated initial codes that were written in the text to resemble their interpretations of short data segments. Codes were generated iteratively across each transcription. After initial coding, analysts independently searched their codes for patterns and, from these patterns, generated an initial list of sub-themes based upon internal and external homogeneity (34). The analysts then met to compare and merge their sub-themes into a single list. Sub-themes were then placed into higher-arching categories (i.e., themes). The themes were then reviewed to ensure that they adequately represented sub-themes, and that sub-themes represented codes. Analysts kept their lists of sub-themes and codes to act as an audit trail. In consideration of the small sample size of the study, the narrow aims, and the strong dialogue by interviewees, the coders aimed to generate a small number of rich themes and sub-themes to enhance the likelihood that the themes achieve informed power (35).

## Results

Interviews were stopped upon interviewing a total of 14 people, because the analysts determined that the surface-level themes achieved informed power. The mean age of the sample was 50 ± 16 years and there were 6 males (42.9%) and 8 (57.1%) females. Regarding race, 6 self-identified as Caucasian; 5 African American, 1 American Indian/Alaskan Native, and 2 were Unknown. Participants were from several different cities across the U.S including Birmingham, AL, Boston, MA, Chicago IL, Denver, CO, and New York, New York.

### Themes

The resultant themes incorporated rich participant feedback related to *social support, self-regulation* motivational factors, *lifestyle changes*, and factors that affected *participation*. These themes are described below and presented with their supporting sub-themes and codes in Table 1.

**Table 1:** Resultant themes.

**Theme**	**Sub-themes**	**Supporting text**
Social support	Intimate community for personal growth through peer support	“We all had different experiences or different things that we thought worked and didn't. There were a lot of people that shared some things. Because we all had some kind of limitation. If one person said I couldn't do this because I didn't know how or I didn't understand this part, there might have been someone that said there's something adaptive that you can use that they didn't know about.” Participant 3 “It made me feel like I was trying to give something back in a small way.” Participant 7 “I loved the other mentor participants. They were all sweet and nice. It was nice to be in with a group of people who were kind of all in the same – like had the same goals or similar goals with wanting to change their lives or help out people and learn something from the mentor program. So it was cool to be around like-minded people…I really liked the community aspect of it, meeting the new people and having the small groups.” Participant 1 “There were some people in the group that were very vocal but there's also some people that were very encouraging. You know, like by telling their story. They were encouraging people.” Participant 6
	Socialization and relationship building were enjoyable aspects of MENTOR	“I liked everybody. We had a good time. Like I said, there was one women and she lives here in _____. She and I did and still do our workouts together. We did everything just like we were instructed. We did everything. We still talk to each other at least once a week.” Participant 3 “I made friends with a few (peers) and we of course conversed and talked about just life stuff after classes–it was a little mentor chat thing.” Participant 2
Self-regulation	Program components were holistic, leading to personalized learning and enjoyment.	“I really enjoyed it, because it was all-encompassing. You know, it got everything. It covered the exercise part of it, the nutrition part of it, the mind and body wellness part of it, and then at the end, a coach to walk you through all of those.” Participant 11 “Well, just the scope of things that they covered all the way from mindfulness to nutrition to exercise, and overall.” Participant 6 “Well, the nutrition was very educational for me. I learned a lot. At the time I was trying to make a health switch and the nutrition is I also liked—they always had said reach out to us after class, and I liked that she was able to help me even one-on-one when I had asked about some nutrition changes that I wanted to make in my life with what I wanted to eat especially as an injured person now and my life being different. She was very helpful with that… It (the nutrition program) opened my eyes to things that I didn't really know before I took those classes.” Participant 1
	Online program increased access to improve health through exercise	“I can I tell when I was sitting at home my shoulders were getting really tight and…and I was just like getting bored just sitting at home so I'm kind of glad they did this. So I can get at least a workout in and not have to go to the gym. So I think that home workout was really awesome.” Participant 12
	Shared lifestyle goals increased motivation	“It was nice to be in with a group of people who were kind of all in the same – like had the same goals or similar goals with wanting to change their lives or help out people and learn something from the mentor program. So it was cool to be around like-minded people…I really liked the community aspect of it, meeting the new people and having the small groups.” Participant 1
Lifestyle changes	Improved psychological wellbeing following mindfulness coaching	“It (mindfulness coaching) was very calming. She (the instructor) actually made a comment about something and I told her about me wanting to do certain things and multitask. And she said that's a misconception: multitask. You think you're multitasking, but you're not. You're still only doing one thing at a time.” Participant 3 “It just helps me to, you know, not get all worried about things in the future that I have no control over.” Participant 8 “It definitely gave me tools. I have never really done meditation or mindfulness or the idea that pain is sometimes in your head. That was definitely helpful, especially where I was in like my injury. I was only about at two years then. So definitely helped with today being able to be more mindful: overthinking it or knowing how to meditate when I am meditating through my pain now.” Participant 1
		“Yeah, so…it (mindfulness coaching) really kept my mind clear. The classes were like amazing and like, especially the mindfulness. It just kind of kept me like not going insane, you know, just sitting at home. It made me feel like relaxed a little bit and, you know, and not think about a whole lot so I was really relaxed.” Participant 12
	Maintenance of healthy decision-making behaviors	“It gave me a lot of information that I'm utilizing right now (6 months later) to improve my quality of life.” Participant 2 “I was wanting to start eating healthier and just doing more things that –eat healthier, exercise more, and the class really helped me gear into doing that for the rest of the year” Participant 1 “It definitely gave me tools. I have never really done meditation or mindfulness or the idea that pain is sometimes in your head. That was definitely helpful, especially where I was in like my injury. I was only about at two years then. So definitely helped with today being able to be more mindful: overthinking it or knowing how to meditate when I am meditating through my pain now.” Participant 8 “The physical benefits from the nutrition class, I've eaten better and I eat more vegetables and things like that. Like I said, I got this food service now so I can eat a little healthier. I was eating like TV dinners a lot. Now, prepping the meals is kind of hard for me because I'm in a chair and my kitchen isn't set up for somebody who's in a chair. But other than that, I had an excellent education and I made changes.” Participant 9 “Yeah, it benefitted me, especially in the nutrition, because I am more likely to look for nutritional foods and not be as carefree as I once was. And mindfulness, I definitely reflect back on that a lot and go back to it. I don't practice as much as I would like to because I am distracted easy, but I do try to recall what I learned in class and impart that into my life one way or the other, either when I'm trying to get some rest or just take time out for myself. And then the exercise, I'm still working on my dedication to that, but I know I have to do it.” Participant 10
	Desired post-program support	“They didn't say you could they just said...and if they did open it up to people that have already gone through the program, I would participate in it again.” Participant 11 “It was really fun. I really wish it would have went a little bit longer than eight weeks or six weeks whatever it was.” Participant 12 “For me, the classes could have been longer… It ended so soon.” Participant 1 “Then the exercise program I really enjoyed and I wish they would have been able to continue it.” Participant 10
Participation	Tailored approaches from knowledgeable, personable coaches contributed to high program quality	“To me, it (the program) was well organized. The staff that was a part of it, they were on their A game every session. They led with a lot of confidence.” Participant 3 “I liked the instructor, because she had so much knowledge. The way she conveyed that knowledge to us was very helpful for me. Sometimes people think that you can grasp things quickly, but I am a slow learner on some things, and the way she spoke to us and the way she explained things, it was very helpful for me.” Participant 10 “I liked the fact that she...you know, we were all different ability levels. And if you were in a chair, she would do the movement for people in chairs. And then if you had the ability to stand, she would do the movement for people who could stand. I liked the different demonstrations for ability levels.” Participant 10
	Class scheduling influenced program attendance	“The only thing that was difficult about it was that you guys are two hours or an hour-and-a-half outside my time zone, so I couldn't do any of the mindfulness because the lady had it at 9 and I _____ from 8 until noon. You know what I'm saying? So I didn't get to do any of the mindfulness which was disappointing.” Participant 4 “It wasn't that it was too much. It was just too much with my job and coordinating the times and stuff.” Participant 7
	Required more suitable exercise adaptations for quadriplegia	“That was another area that I felt like the study was not really – it was a little bit broad from the standpoint of like a lot the exercises a quadriplegic couldn't do and if they could maybe like be a little bit more—if you're at this level of function, here's a different type of exercise you could try.” Participant 7 “I thought the exercises were relevant for a handicapped person, so I thought the exercises were good.” Participant 8 “It's not a dislike but my least favorite was the exercise part. Because I have some limitation in my arms and though it was, they adapted everything for me but it was still kind of…just…not…it was…I don't know how to describe it. It was…I didn't feel like I was getting…like it wasn't really feeling like I was doing the exercise or yoga. It was just harder for me…Harder because of my limitation, you know, arm limitations and they adapted, you know, they said if you can't move your arm...it just…they did everything they could. I didn't hate it or dislike it was just…not my number one part.” Participant 11
	Some preferred in-person classes to enhance engagement	“I could picture that the program that you were executing, doing it in-person would be 80% more effective than trying to do it remotely... I think even if you would have gotten the participants in the room and then had the instructors remote that would have been much more effective than trying to have everybody remote. I will tell you that I think it's hard, the program you executed was really difficult to keep peoples' attention and to get them really 100% focused on what you're doing.” Participant 9 “I think online mindfulness is really hard for me.” Participant 4

*Social support* was an enjoyable component of MENTOR. Participants reported that the group-based classes provided a valuably intimate sense of community with other members. Value was created by either receiving or providing verbal support. Examples of these interactions included recommending adaptations for various activities or overcoming other physical or emotional life challenges. Small groups fostered in-depth communication and relationship building, which led to some participants creating peer friendships and bonds outside of the class after the program ended. Groups were balanced by participants who were vocal and others who were less conversive.

*Self-regulation* motivational factors were attributed to the holistic nature of the program, the accessible online platform, and shared lifestyle goals. The program offered a variety of opportunities for participants to self-select classes or certain components that they felt best fit their personal needs. Some participants found themselves enjoying and attending all the classes across the 8-week program, which was a substantial time investment (5 hr/week for 8 weeks). Some participants reported that nutrition was the most helpful toward improving their lifestyle, while others reported that the mindfulness component was most beneficial. Since MENTOR was provided through an online platform, participants appreciated that the classes could be conveniently attended without requiring transportation to an onsite facility. Moreover, self-improvement goals were often shared by other members, which strengthened their motivation to attend the classes.

*Lifestyle changes* were a positive benefit of participation in MENTOR. Participants reported that they adopted healthy behaviors and sustained these behaviors long after the program ended. Participants made valuable changes to their nutritional behaviors to eat healthier. Nutritional guidance was provided to participants at a depth they had not expected or previously received. Mindfulness strategies were novel to most participants and helped them cope with pain and other life stressors. Most importantly, nutrition and mindfulness strategies were sustained after the program ended.

*Participation* was affected by several factors. Regarding facilitating factors, participants reported that the program content was perceived to be of high quality, which was attributed to the professional and knowledgeable coaches. Coaches were perceived as prompt, knowledgeable of disability, helpful, and confident. Additionally, participants reported that exercises were generally well tailored to their functional and cognitive abilities. Regarding barriers to participation, some participants with more severe functional impairment (e.g., quadriplegia) reported that exercises were not suitable to their functional ability. Specifically, people with tetraplegia desired more in-depth adaptations for their arms that would increase the ease of difficulty with performing similar movements as others in the class, while also exhibiting sufficient effort or work to provide a physiologic health benefit. Additionally, some participants reported that they would like the option to attend in-person classes. These participants did not feel engaged through telecommunication. Some participants' ability to attend the classes was restricted by their availability (e.g., worked a full-time job or had family responsibilities). To remedy this, participants recommended more options for class times. Even though the daily class regime was provided to participants as a way of allowing them to choose various components that could fit into their daily routine, participants were disappointed by having to miss class components.

## Discussion

This retrospective evaluation of a holistic, community-based program found that people with SCI value comprehensive online wellness for providing a sense of peer-support and promoting sustainable, health-enhancing behaviors. Participants also reported improved psychological well-being. This finding is similar with benefits reported in a scoping review of 20 qualitative clinical studies examining wellness interventions for people with SCI (36). Of note, the scoping review recommended that clinical studies of wellness should include a broad understanding of well-being and be more accessible to people with SCI, traits inherent in the MENTOR program. Most of the previous studies were conducted in clinical settings or required in-person supervision or visitation. MENTOR provided a fully remote, no-cost program across the nation for people with SCI who had internet access. The program included daily classes that participants could choose and prioritize based on their needs and interests, and the group-based structure and one-on-one coaching promoted peer-supported mentoring.

Quantitative, evidence-based wellness programs for people with SCI are sparse. One study published in 2017 descriptively reported that a wellness program for 45 people with SCI may have improved pain management, depression levels, and exercise behavior, with reductions in time spent watching television (37). Another study demonstrated that 23 people with SCI who participated in a wellness program had statistically significant improvements in health behaviors and self-efficacy, as well as fewer and less severe secondary health condition compared to a control group (*n* = 20). One study published in 2016 found that a more medically focused wellness intervention improved function, mood, and quality of life (38). The study further suggested that a wellness program could potentially produce a financial return on investment over a 2-year period (38). MENTOR demonstrated quantitative improvements to exercise behavior, nutrition, sleep, core values, self-care, hobbies, and contribution to society/community, relationships, and overall wellness with a general cohort of people with physical disabilities (31). However, further evidence is needed to confirm these benefits among specific subgroups, such as people with SCI.

Of note, qualitative findings demonstrated that three components, namely, peer-support and relationship building, comprehensive wellness options, and expert coaches, were well received by people with SCI. Peer-support is a strong facilitator of wellness programs (36) and is even a strongly desired trait of exercise only programs (39). Future programs should aim to produce more inclusive class times, provide specific exercise adaptations for people with limited arm function, and provide in-person options for attendance (e.g., peer meetings with remote instructors).

This study had several limitations. First, the evaluation was conducted retrospectively. Some participants completed the program long after others (durations ranging from 5 to 18 months), which could have affected their ability to recall specific aspects or benefits of the program. Second, the sample included a slightly larger number of females than males, which may not be representative of SCI, which generally has a higher incidence among males (40). Third, we were not able to report the lesion levels of participants. Fourth, participants were required to have access to the internet which may not be feasible for some people with SCI. Fifth, given the small sample size, the study findings are not generalizable but, instead, should be transferable to similar settings and traits of those involved.

## Conclusion

The need among people with SCI to improve health and prevent/manage secondary conditions can be greatly enhanced by increasing their access to the social determinants of wellness. As a government-sponsored program, MENTOR has the capacity over the next 5 years to reach more and more people with SCI across the U.S. in an effort to provide quality programs and services to this underserved population. The social determinants of wellness can have a profound effect on improving health and reducing the risk of age-associated chronic diseases in people with SCI.

## Data Availability Statement

The raw data supporting the conclusions of this article will be made available by the authors, without undue reservation.

## Ethics Statement

The studies involving human participants were reviewed and approved by University of Alabama at Birmingham Institutional Review Board. Written informed consent for participation was not required for this study in accordance with the national legislation and the institutional requirements.

## Author Contributions

RY, MC, and CL were responsible for conducting the interviews. BL and MC analyzed the data. TS, JR, and BL created the initial draft of the manuscript. H-JY and JW created the second manuscript draft. All authors contributed to the final manuscript draft.

## Funding

Funding for this study was provided by the Centers for Disease Control and Prevention (CDC), Division of Birth Defects and Developmental Disabilities (NCBDDD), Disability and Health Promotion Branch, Grant #NU27DD000022.

## Conflict of Interest

The authors declare that the research was conducted in the absence of any commercial or financial relationships that could be construed as a potential conflict of interest.

## Publisher's Note

All claims expressed in this article are solely those of the authors and do not necessarily represent those of their affiliated organizations, or those of the publisher, the editors and the reviewers. Any product that may be evaluated in this article, or claim that may be made by its manufacturer, is not guaranteed or endorsed by the publisher.
